# Spatial yield gains in empty-row optimized rice–crab co-culture are linked to *nifH*-driven nitrogen compensation in border rows

**DOI:** 10.3389/fpls.2025.1607596

**Published:** 2025-08-15

**Authors:** Tiexin Yang, Dandan Jin, Liqiang Dong, Liang Ma, Zhengyan Pan, Zhiqiang Li, Fuyu Sun, Xiaosen Sun, Lei Yu

**Affiliations:** ^1^ Rice Research Institute, Liaoning Academy of Agriculture Science, Shenyang, China; ^2^ Plant Nutrition and Environmental Resources Research Institute, Liaoning Academy of Agriculture Science, Shenyang, China; ^3^ Plant Protection Research Institute, Liaoning Academy of Agriculture Science, Shenyang, China; ^4^ Chifeng Product Quality and Safety Center of Agricultural and Livestock, Chifeng, China

**Keywords:** rice-crab, nitrogen cycle gene, boundary yield, rice yield, soil nitrogen fixation

## Abstract

**Introduction:**

The rice–crab coculture system is ecologically sustainable with efficient resource utilization, but the soil nitrogen cycling mechanisms underlying yield limitations in different coculture models remain unclear. Here, we aimed to identify yield-limiting factors by comparing rice productivity between the conventional rice–crab coculture model (CK) and an optimized model (12 rows cultivated-1 row empty, ERC-12). We hypothesized that ERC-12 enhances crab activity in empty rows, thereby stimulating nifH-mediated soil nitrogen fixation to offset yield losses caused by reduced planting density.

**Methods:**

Field experiments were conducted in Panjin, Liaoning Province, during 2023–2024 using two japonica cultivars, Yanjing 939 (YJ939) and Yanfeng 47 (YF47). Plots were arranged in CK and ERC-12 patterns; the latter was spatially divided into boundary (PB), intermediate (PM), and central (PC) zones. Yield components, aboveground dry matter (ADM), and nitrogen (N) accumulation were measured. Soil NH₄⁺-N, NO₃⁻-N, and other nutrients were analyzed at tillering and heading stages. Expression of nitrogen-cycling genes (nifH, nirK, nirS, etc.) was quantified by qPCR.

**Results:**

Our results showed that although ERC-12 increased per-plant yield via marginal effects in the boundary zone (PB), total yield decreased by 4.06%-5.20% compared to CK, primarily due to yield losses in the intermediate zone (PM) and empty rows. Correlation analysis revealed that the PB zone in ERC-12 had significantly higher soil ammonium nitrogen (NH+ 4-N) content and elevated expression of the nitrogen-fixing gene nifH (p < 0.01), which promoted aboveground dry matter accumulation and yield—consistent with enhanced biological nitrogen fixation under crab activity. In contrast, the PM zone suffered from nutrient competition and reduced activity expression of key nitrogen-cycle genes such as nifH, nirK, and nirS, becoming a key yield-limiting factor.

**Disscussion:**

ERC-12 partially compensates for yield losses through elevating soil nifH expression, which enhances NH4 +-N supply in the PB zone. To further improve ERC-12 yield, targeted strategies should be applied to optimize rice population structure in the boundary zone, the intermediate zone, and the central zone (PC), alleviating nutrient limitations in the PM zone while maintaining the boundary yield advantage.

## Highlights

- ERC-12 increased per-plant yield via marginal effects in the boundary zone.
- The soil $NH_{4}^{+}-N$ content was the main factor affecting yield.
- The marginal effects of the boundary zone of ERC-12 increased soil $NH_{4}^{+}-N$ content.
- The ERC-12 model enhanced the soil nitrogen fixation and *nifH* expression level.

## 1 Introduction

Amid relentless global population growth and increasingly frequent extreme weather events, food security remains a paramount concern (Faroop et al., 2022; Janni et al., 2024). As a staple food for more than half of the world’s population, rice (*Oryza sativa* L.) plays a pivotal role in maintaining global food security, with sustainable productivity being vital for meeting future nutritional demands (Yuan et al., 2022a; Bin Rahman and Zhang, 2023). However, conventional rice system is under mounting pressure from excessive synthetic-fertilizers, which not only reduces productivity but also exacerbates soil degradation under intensified agriculture (Hashim et al., 2024; Mukhopadhyay et al., 2021). Therefore, address these intertwined challenges of agricultural sustainability and environmental preservation, there is an urgent need to develop an innovative cultivation model that balances productivity with ecological conservation.

The rice**–**crab coculture model, an ecologically sustainable cultivation system, integrates “rice cultivation” and “crab aquaculture” to establish a dual production ecosystem characterized by “one field for dual production and one pond for dual harvests” (Hu et al., 2015; Zhao et al., 2020; Dong et al., 2021). This integrated approach offers multiple benefits: it enhances cultivated land utilization efficiency while promoting biodiversity conservation and improving the ecological balance of paddy ecosystems (Yuan et al., 2022b). When compared with the conventional rice monoculture model, the coculture model showed crabs prey on pests and weeds in the paddies, and their metabolic byproducts provide additional organic fertilizer for rice growth, resulting in substantial reductions in chemical fertilizer and pesticide application throughout the rice growth cycle (Chen et al., 2021). Through four decades of agricultural innovation in China, various regionally adapted cultivation techniques for the rice**–**crab coculture model have emerged, including the “Liaoning Panshan model” in Liaoning Province, the “Ningxia Rice**–**Crab Model” in Ningxia Hui Autonomous Region and the “Jilin Crab Aquaculture Technology Model” in Jilin Province (Wang et al., 2013; Qu et al., 2021; Wang and Wang, 2007).

As a major japonica rice-producing region in northern China, Liaoning Province offers unique geographical advantages and superior ecological conditions that facilitate the implementation of integrated rice**–**crab farming systems (Sun et al., 2017; Wang and Feng, 2024). Building upon the foundational “Panshan Model”, the optimized rice**–**crab coculture model known as the “12**–**rows**–**cultivated**–**1**–**row**–**empty model” optimizes both the rice planting structure and crab living environment, strategically balancing rice productivity with crab biomass output and maximize economic returns for farmers (Ma et al., 2023). Although our preliminary research showed that the comprehensive economic benefit of paddies under the optimized model was CNY 3,000 per hectare higher than under the conventional model, yield stability remains a critical constraint on a large scale (Ma et al., 2023; Chen et al., 2021). Under increasing food security pressures, unlocking the rice yield potential of this optimized model has become imperative for the regional scalability. In years of rice-crab co-culture production practice, we have observed an interesting phenomenon: despite a 7.69% reduction in rice planting area in optimized rice**–**crab coculture model compared to the conventional rice**–**crab coculture model, the decrease in rice yield is relatively small (Ma et al., 2023; Zhao and Yang, 2024). Based on this observation, we speculate that there may be a mechanism in optimized rice**–**crab coculture model that partially compensates for the potential yield loss caused by the reduced planting area. Additionally, our previous studies have shown that significant changes in soil organic matter content, microbial diversity and richness under the rice**–**crab coculture model (Ma et al., 2024b), and the activity of crabs has been confirmed to promote soil biological nitrogen fixation by enhancing soil aeration (Li et al., 2024b). This may lead to spatial variations in soil nitrogen supply within ERC**–**12, thereby causing spatial yield heterogeneity (Ma et al., 2023). Current research predominantly focuses on the economic evaluations and agronomic traits of the model (Ma et al., 2021, 2023, 2024a), while the soil**–**microbial mechanisms driving yield differentials remain unclarified.

Therefore, in this research, we investigated the underlying causes of yield differences between the conventional and optimized rice**–**crab coculture models. We proposed three hypotheses regarding the spatial yield heterogeneity in ERC-12 yield: (1) In crab**–**inhabited areas, their activities significantly upregulate the expression of the soil nitrogen-fixing functional gene *nifH* by enhancing soil aeration and organic matter input, thereby increasing NH_4_^+^-N supply; (2) The spatial zoning of ERC-12 (boundary zone, intermediate zone and central zone) leads to nitrogen supply heterogeneity; (3) *nifH* ***–*** mediated biological nitrogen fixation can partially compensate for yield losses attributed to empty rows. We analyzed the yield and yield components of the two models, as well as dynamic changes in soil nutrients and their spatial distribution, to explore yield differences between the models from a nutrient supply perspective. Additionally, we detected the expression levels of key genes involved in soil nitrogen cycling across different ERC**–**12 zones to reveal the soil microbial mechanism driving spatial yield heterogeneity in the ERC**–**12 model from the perspective of soil nitrogen cycling.

## 2 Methods and materials

### 2.1 Experimental site

The field experiments were conducted during the 2023–2024 growing seasons in Panshan County, Panjin City, Liaoning Province, North China (41°19’34”N, 122°3’52”E), within the Liaohe River Delta coastal saline plain. Rice production here relies primarily on river irrigation. Since the 1980s, the rice production system in this region has been gradually transitioning from monoculture to a rice**–**crab coculture. Currently, over 95% of rice produced here is associated with the rice**–**crab coculture system, with an average annual cultivation area of 400,000 ha.

### 2.2 Experimental design

The rice varieties used were Yanjing 939 (*Oryza sativa* L. subsp*. japonica*, YJ939) and Yanfeng 47 (*Oryza sativa* L. subsp*. japonica*, YF47), the predominant cultivars in this area, with a whole growth period of approximately 160 days for both varieties. Rice was sown on April 10, 2023, and April 8, 2024, and transplanted on May 23, 2023, and May 24, 2024, using mechanical transplanting at a density of 30 cm×18 cm and 4–5 seedlings per hill. Two rice**–**crab coculture models were established: (1) the conventional rice**–**crab coculture model (CK), featuring continuous rice planting without interrow ditches; and (2) the optimized 12**–**rows**–**cultivated**–**1**–**row**–**empty model (ERC-12), with alternating blocks of 12 rice rows with 1 ditch row. Each model occupied a planting area of approximately 1 ha, with three 10m×10m replicate plots randomly selected as for field investigations and sampling.

**Figure 1:**
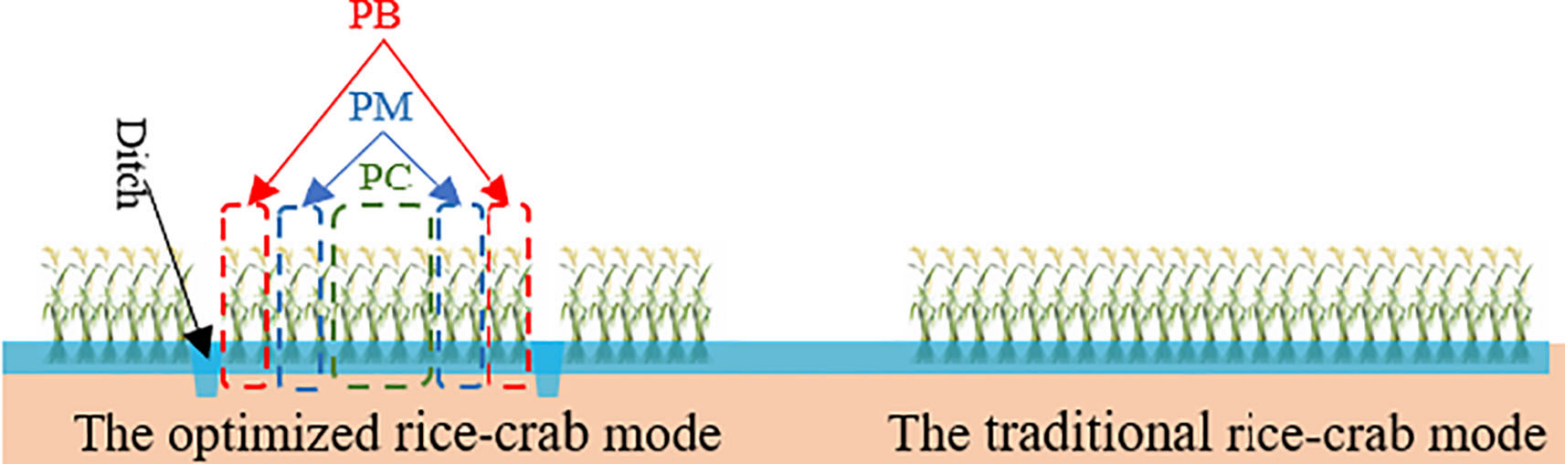
The conceptual diagrams of optimized rice-crab coculture model (ERC-12) and traditional rice-crab coculture model.

Our previous research showed that in ERC-12, rice yield per hill from exhibited a trend of first decreasing then increasing from the edge rows toward the interior. Based on the spatial arrangement of rice hills, significant yield differences were observed among the boundary rows, intermediate rows and central rows (S1). Thus, each 12**–**rows rice block in ERC-12 was divided into three spatial zones according to its proximity to the adjacent ditch: the boundary zone (PB), the intermediate zone (PM), and the central zone (PC) (**Figure 1**).

Following established management practices for the rice**–**crab coculture model (Zhou et al., 1995; Zhao and Yang, 2024), a basic fertilizer application of N 243 kg/ha, P_2_O_5_ 117kg/ha, and K_2_O 135 kg/ha was applied once, with no additional fertilization throughout the growth cycle. Juvenile crabs (*Eriocheir sinensis*) were stocked in the paddy at a density of 75 kg/ha before June 10, with feeding initiated on the subsequent day. Daily rations maintained at 3**–**5% of crab biomass. No pesticides were used during rice growth; instead, bait and UV insecticide lamps were employed for pest control. A water layer was maintained throughout the rice growth period, with depths of approximately 3–5 cm at transplanting, ≤10 cm at tillering, ≤15 cm at jointing–booting, and ≤20 cm at grain filling.

### 2.3 Yield indices

#### 2.3.1 Dry matter weight and nitrogen accumulation in the aboveground parts of rice

At the full tillering, full heading, and maturity stages of rice, 3 consecutive plants were sampled from CK and the PC, PM and PB zones of ERC-12. Aboveground tissues were harvested, rinsed with distilled water, deactivated at 105°C for 30 min, and then dried at 80°C to constant weight. After drying, the aboveground dry matter (ADM) was measured. Following mass determination, each of dried sample was ground, and the nitrogen content of the aboveground tissue was analyzed using the Kjeldahl method as described by Sheng et al. (2022).

#### 2.3.2 Yield and yield components of rice

Before being harvested, 3 consecutive plants were sampled from CK and the PC, PM and PB zones of ERC**–**12 to measure effective panicle number per hill, the spikelets per panicle, 1000-grain weight and seed setting rate. Grain moisture was standardized to 14.5% to calculate theoretical rice yield (Dong et al., 2023).

Mechanical harvesting was used to collect all rice from the CK and ERC-12 production areas. Harvesting was timed based on local climate and rice maturity, finishing by October 20 each year. Actual rice yield was calculated after converting to grain moisture to 14.5%.

#### 2.3.3 Soil nutrient and soil nitrogen cycling functional genes

At the full tillering and full heading stages, soil samples were collected from CK and the PC, PM and PB zones of ERC**–**12. For each plot, 6 soil samples were randomly collected, with 3 samples pooled to form one biological replicate (two biological replicates per plot). Soil samples were stored at -80°C for subsequent analysis of soil nitrogen cycle functional genes (Li et al., 2024a). Gene expression levels were normalized using the 16S rRNA gene as an internal reference to account for variations in microbial community abundance. For relative expression analysis via the ΔΔCt method, PCR efficiency (90–110%) was first validated using a standard curve generated from 10^−1^ to 10^−6^ serial dilutions of target gene plasmids. Expression values were then normalized based on total RNA yield per gram of dry soil (quantified by Nanodrop and adjusted to 1 μg/μL) to mitigate inter-sample nucleic acid concentration biases. qPCR primer sequences and annealing temperatures are detailed in S2.

Pooled biological replicates (by combining two replicates per plot) were used for soil physicochemical property analysis. Soil samples were divided into two subsamples: the first stored at 4°C for nitrate nitrogen content ( $NO_{3}^{-}-N$ ) and ammonium nitrogen content ( $NH_{4}^{+}-N$ ), and the second air-dried naturally for measuring pH, organic matter (OM) content, total nitrogen (TN) content, total phosphorus (TP) content, total potassium (TK) content, available nitrogen (AN) content, available phosphorus (AP) content and available potassium (AK).

### 2.4 Statistical analysis

Data were organized and managed using Microsoft Excel 2019 (Microsoft Corporation, Redmond, WA, USA). All primary statistical analyses were performed using SPSS Statistics 22.0 (IBM Corp., Armonk, NY, USA). A two-way factorial ANOVA was used to evaluate the main and interactive effects of year, variety, and treatment after confirming normality (Shapiro-Wilk) and homoscedasticity (Levene’s test). When interactions were non-significant, one-way ANOVA was applied to assess individual factors. Tukey’s Honestly Significant Difference (HSD) test was employed for *post hoc* comparisons to control for Type I error across multiple comparisons (α = 0.05). Pearson correlation was applied to normally distributed variables, and Spearman’s rank correlation to non-normal data, with significance declared at p < 0.05 and p < 0.01, respectively. Repeated-measures ANOVA or paired-sample t-tests were used for within-plot measurements across growth stages; independent-sample t-tests were reserved for unpaired comparisons. Partial least squares structural equation modeling (PLS-SEM) was performed with SmartPLS 4.1 (SmartPLS GmbH, Boenningstedt, Germany) to investigate complex interrelationships among variables. Data visualization was carried out using ORIGIN 2024b (OriginLab Corporation, Northampton, MA, USA).

## 3 Results and analysis

### 3.1 Yield and yield components of rice

**Figure 2:**
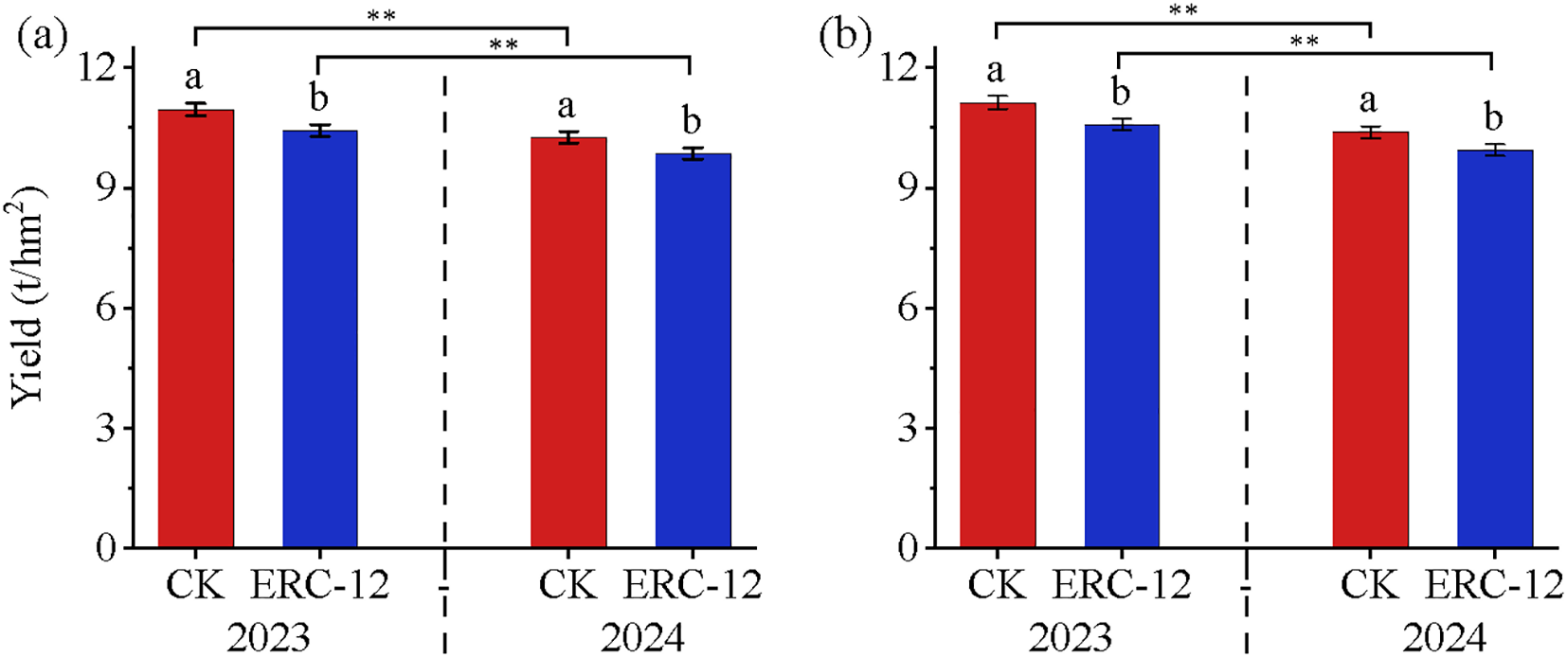
The yield of YJ939 and YF47 in 2023 and in 2024, respectively. **(a)** the yield of YJ939, **(b)** the yield of YF47. CK, the traditional rice-crab co-culture model. ERC-12, the optimized rice-crab co-culture model. Different letters show significant difference between samples at α = 0.05 (Tukey’s HSD). ** show significant difference between samples α = 0.05 (two-tailed). Three biological replicates and the average deviation is used for the error line.

**Figure 3:**
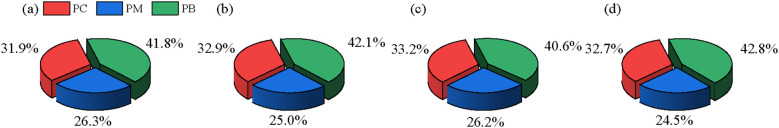
Contribution of PC, PM and PB to ERC-12 yield. **(a, c)** indicate YJ939 in 2023 and in 2024, respectively. **(b, d)** indicate YF47 in 2023 and in 2024, respectively. PB, boundary zone; PM, intermediate zone; PC, central zone.

To identify factors limiting rice yield in a rice**–**crab coculture system, we analyzed yield and yield components across the two models, different years, varieties and cultivation zones. A comparison of actual yield showed that CK yields were significantly higher than those of ERC-12 (**Figure 2**), with significant annual differences (p < 0.01). In 2023, ERC**–**12 yields for YJ939 and YF47 were 10950 kg/ha and 11130 kg/ha, respectively, corresponding to 4.99% and 5.20% decreases relative to CK. In 2024, ERC**–**12 yields were 10260 kg/ha of YJ939 and 10390 kg/ha of YF47, with reductions of 4.06% and 4.53% compared to CK. Analysis of yield components (**Table 1**) indicated that effective panicle number per hill, spikelets number per panicle, 1000-grain weight and seed setting rate were the highest in the PB zone and lowest in the PM zone for both varieties, while PC zone components were comparable to CK. Statistical analysis revealed significant variety × treatment interactions for panicle number and 1000-grain weight (p < 0.05), and significant year × variety interactions for the seed-setting rate. The ERC-12 production area consists of PB, PM, PC, and empty rows, with PB, PM, and PC being the main contributors to the yield. By analyzing the yield components of these zones, the theoretical yield per hole of each zone was calculated and used to evaluate their contribution rates to the actual yield of ERC-12 (**Figure 3**). The results showed, PB was the primary contributor (40.6–42.8%), followed by PC (31.9–33.2%) and PM (24.5–26.3%), with this hierarchy consistent across 2023 and 2024.

**Table 1:** The yield components of YJ939 and YF47 in 2023 and in 2024, respectively.

Year	Varieties	Treatments	Effective panicles (/hill)	Spikelets numbers (/panicle)	1000-grain weight (g)	seed setting rate (%)
2023	YJ939	CK	15.45 ± 1.15ab	136.28 ± 10.12a	25.24 ± 0.30b	87.42 ± 1.03b
		PC	15.68 ± 0.23ab	134.46 ± 1.98a	25.09 ± 0.14b	87.67 ± 0.50b
		PM	13.85 ± 1.03b	129.46 ± 9.61a	24.18 ± 0.14c	84.15 ± 0.48c
		PB	16.31 ± 0.26a	142.46 ± 3.16a	26.64 ± 0.31a	91.64 ± 1.08a
	YF47	CK	22.85 ± 0.73b	112.28 ± 3.73ab	23.18 ± 0.13c	88.76 ± 0.96b
		PC	22.07 ± 0.55b	109.85 ± 3.18ab	23.69 ± 1.40b	89.68 ± 1.26b
		PM	19.31 ± 1.02c	107.63 ± 2.36b	21.28 ± 0.13d	87.23 ± 0.77b
		PB	25.63 ± 2.46a	116.32 ± 1.56a	24.15 ± 0.14a	93.61 ± 0.83a
2024	YJ939	CK	14.43 ± 1.14ab	136.87 ± 11.05a	25.27 ± 0.22b	87.37 ± 0.69b
		PC	14.58 ± 0.38ab	133.83 ± 2.10a	25.02 ± 0.13b	87.54 ± 0.42b
		PM	13.43 ± 0.98b	129.12 ± 9.75a	24.16 ± 0.04c	83.96 ± 0.70c
		PB	16.10 ± 0.23a	142.62 ± 4.04a	26.66 ± 0.31a	91.64 ± 1.45a
	YF47	CK	21.82 ± 0.75b	111.38 ± 2.78ab	23.21 ± 0.16b	86.97 ± 0.84b
		PC	21.71 ± 0.50b	109.79 ± 3.47b	23.70 ± 0.14a	86.29 ± 0.80b
		PM	18.94 ± 0.91c	108.26 ± 1.91b	21.25 ± 0.17c	84.80 ± 1.11b
		PB	24.17 ± 1.00a	117.20 ± 1.68a	24.11 ± 0.16a	91.24 ± 1.16a
Analysis of variance						
Years (Y)			**	Ns	Ns	**
Varieties (V)			**	**	**	**
Treatments (T)			**	**	**	**
Y*V			Ns	Ns	Ns	**
Y*T			Ns	Ns	Ns	Ns
V*T			**	Ns	**	Ns
Y*V*T			Ns	Ns	Ns	Ns

Different letters in the same column for the same year show significant difference between samples at α = 0.05 (Tukey’s HSD). ** Show significant difference between samples at α = 0.01 (Tukey’s HSD). Ns, show nonsignificance. PB, boundary zone of ERC-12; PM, intermediate zone of ERC-12; PC, central zone of ERC-12; CK, the traditional rice-crab co-culture model; ERC-12, the optimized rice-crab co-culture model. Mean ± SD, n=3.

### 3.2 Dry matter weight and nitrogen accumulation in the aboveground parts of rice

**Figure 4:**
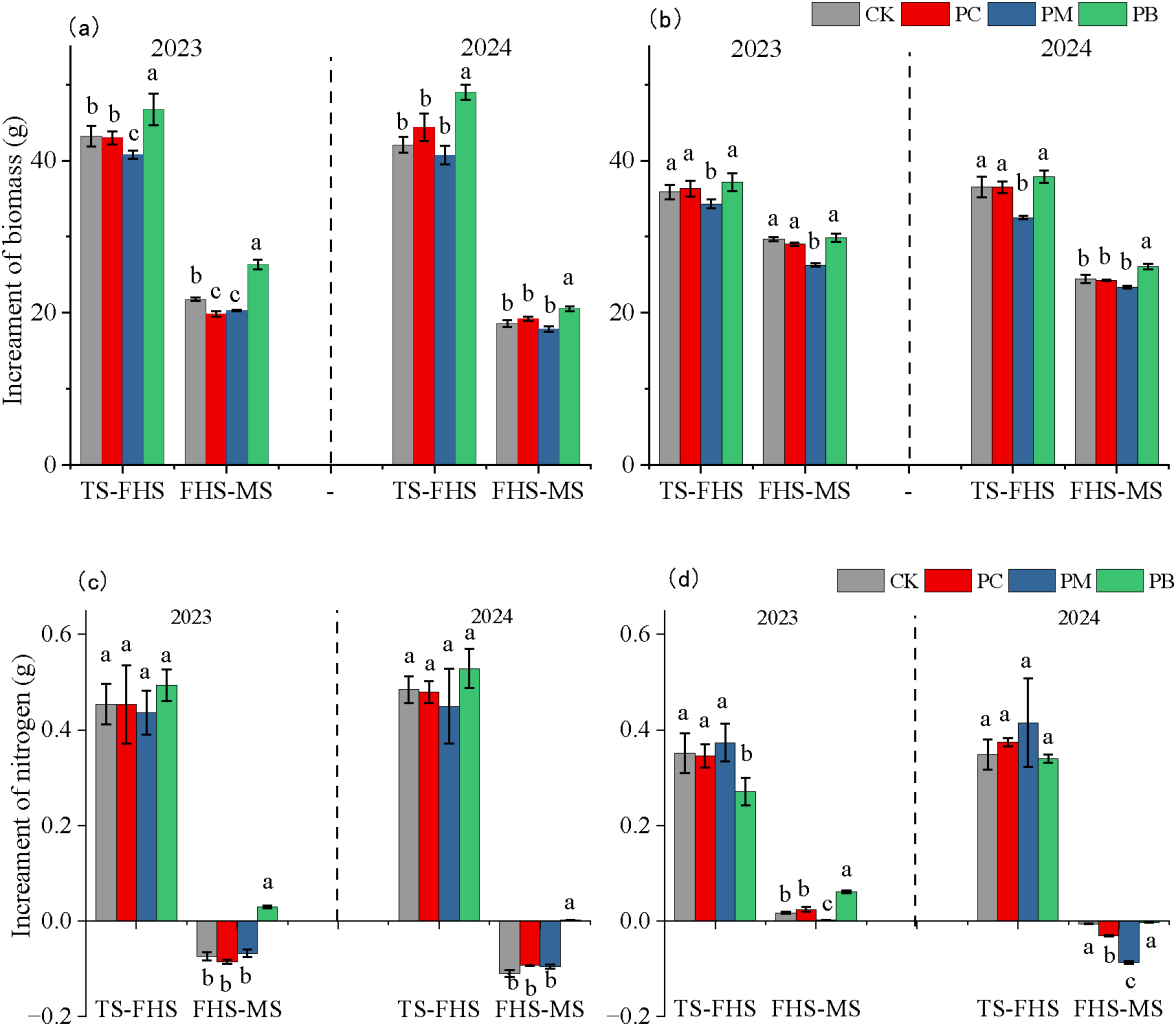
The average increment of dry matter and nitrogen content of aerial rice in different stages. **(a, c)** indicates YJ939, **(b, d)** indicates YF47. Different lowercase letters show significant difference at α = 0.05 (Tukey’s HSD). PB, boundary zone of ERC-12; PM, intermediate zone of ERC-12; PC, central zone of ERC-12. TS, the tillering stage. FHS, the full heading stage. MS, the maturity stage. CK, the traditional rice-crab co-culture model. ERC-12, the optimized rice-crab co-culture model. Three biological replicates and the average deviation is used for the error line.

To identify yield**–**limiting factors, we analyzed aboveground dry matter (ADM) weight and nitrogen accumulation of rice across growth stages, considering different years, varieties, and cultivation zones. As shown in **Table 2**, both YJ939 and YF47 exhibited the highest ADM in the PB zone and the lowest in the PM zone at the full tillering, full heading, and maturity stages. Statistical analysis revealed significant effects on ADM from: (1) year, variety, treatment, and variety × treatment interaction at the full tillering stage; (2) variety and treatment at the full heading stage; and (3) year, variety, and treatment at the maturity stage. Nitrogen accumulation patterns mirrored ADM dynamics across treatments. Significant effects on nitrogen accumulation were observed from variety and treatment at the full tillering and full heading stages, and from variety, treatment, and variety × treatment interaction at the maturity stage. An analysis of ADM and nitrogen accumulation rates during two key periods (full tillering to full heading, and full heading to maturity stage (**Figure 4**) showed that PB consistently exhibited the highest rates, while PM showed the lowest. Varietal differences in nitrogen accumulation were observed that YJ939 in PB zone accumulated nitrogen during the full heading**–**to**–**maturity period only in 2023. In YF47 in PB zone showed nitrogen accumulation during this period in 2023 but not in 2024.

**Table 2:** The aboveground dry matter and nitrogen contents of YJ939 and YF47 per hill.

Years	Varieties	Treatments	Aboveground dry matter (g/hill)			Nitrogen contents (g/hill)		
			TS	FHS	MS	TS	FHS	MS
2023	YJ939	CK	13.22 ± 0.29b	56.46 ± 3.42a	78.24 ± 2.20b	0.3450 ± 0.0119b	0.7989 ± 0.0473ab	0.7253 ± 0.0211b
		PC	12.84 ± 0.22b	55.84 ± 3.70a	75.70 ± 1.49bc	0.3324 ± 0.0117b	0.7860 ± 0.0465ab	0.7012 ± 0.0144b
		PM	11.25 ± 0.31c	52.01 ± 5.69a	72.33 ± 0.99c	0.2849 ± 0.0165c	0.7213 ± 0.0440b	0.6535 ± 0.0104c
		PB	15.11 ± 0.42a	61.86 ± 6.97a	88.20 ± 1.21a	0.3970 ± 0.0228a	0.8905 ± 0.0532a	0.9204 ± 0.0128a
	YF47	CK	18.35 ± 0.64b	54.23 ± 3.45ab	83.23 ± 1.63b	0.4017 ± 0.0344b	0.7530 ± 0.0733a	0.7705 ± 0.0139a
		PC	17.81 ± 0.63bc	53.12 ± 1.73b	82.49 ± 1.62b	0.3943 ± 0.0335b	0.7403 ± 0.0225a	0.7652 ± 0.0150ab
		PM	15.04 ± 0.96c	46.98 ± 1.67b	74.99 ± 1.51c	0.3241 ± 0.0276b	0.6978 ± 0.0188a	0.6998 ± 0.0062b
		PB	23.21 ± 1.46a	60.18 ± 1.74a	87.92 ± 4.81a	0.4992 ± 0.0430a	0.7702 ± 0.0752a	0.8320 ± 0.0468a
2024	YJ939	CK	12.72 ± 0.27b	56.15 ± 1.50ab	76.13 ± 3.64ab	0.3319 ± 0.0117b	0.8163 ± 0.0475ab	0.7064 ± 0.0321b
		PC	12.34 ± 0.20b	56.72 ± 3.06ab	75.95 ± 2.19b	0.3197 ± 0.0113b	0.7988 ± 0.0465ab	0.7061 ± 0.0190b
		PM	10.83 ± 0.30c	51.54 ± 3.04b	69.41 ± 2.00b	0.2740 ± 0.0159c	0.7236 ± 0.0380b	0.6285 ± 0.0176b
		PB	14.52 ± 0.40a	63.49 ± 3.98a	84.04 ± 4.02a	0.3818 ± 0.0223a	0.9102 ± 0.0530a	0.9127 ± 0.0431a
	YF47	CK	17.69 ± 1.21b	53.56 ± 2.48ab	78.65 ± 1.94ab	0.3862 ± 0.0332b	0.7348 ± 0.0191ab	0.7290 ± 0.0193b
		PC	16.81 ± 0.43b	53.12 ± 1.73ab	77.97 ± 4.39b	0.3793 ± 0.0325b	0.7538 ± 0.0052ab	0.7235 ± 0.0393b
		PM	14.37 ± 0.58c	49.38 ± 1.90b	70.33 ± 2.31b	0.3116 ± 0.0268c	0.7267 ± 0.0271b	0.6403 ± 0.0118b
		PB	22.21 ± 1.24a	60.18 ± 2.69a	86.25 ± 2.13a	0.4797 ± 0.0412a	0.8199 ± 0.0547a	0.8163 ± 0.0195a
Analysis of variance								
Years (Y)			*	Ns	**	Ns	Ns	**
Varieties (Y)			**	**	**	**	**	Ns
Treatments (T)			**	**	**	**	**	**
Y*V			Ns	Ns	Ns	Ns	Ns	Ns
Y*T			Ns	Ns	Ns	Ns	Ns	Ns
V*T			**	Ns	Ns	Ns	Ns	**
Y*V*T			Ns	Ns	Ns	Ns	Ns	Ns

Different letters in the same column for the same year show significant difference between samples at α = 0.05 (Tukey’s HSD). *, ** show significant difference between samples at α = 0.05 and 0.01, respectively (Tukey’s HSD). Ns, show nonsignificance; PB, boundary zone of ERC-12; PM, intermediate zone of ERC-12; PC, central zone of ERC-12; CK, the traditional rice-crab co-culture model; ERC-12, the optimized rice-crab co-culture model. Mean ± SD, n=3.

### 3.3 The soil nutrient

To assess the nutrient supply capacity of the two rice-crab coculture models, soil physicochemical properties were analyzed across different zones. As shown in **Tables 3**, **4**, changes in soil physicochemical properties were consistent between YJ939 and YF47 at the full tillering and full heading stages. The soil pH remained relatively stable. Compared with the full tillering stage, soil pH of YJ939 slightly decreased at the full heading stage, while no significant difference was observed for YF47. The OM content varied with treatment and growth stage. At the full tillering stage, the OM contents in the PB and PM zones were higher than those in PC zone and CK. Conversely, at the full heading stage, OM contents in the PC zone and CK exceeded those in PB and PM zones. TP and TK contents exhibited significant changes only in a few specific treatments. For all treatments, AP and AK contents were significantly higher at the full heading stage than at the full tillering stage.

**Table 3:** The soil physicochemical properties of YJ939.

Years	Stages	Treatments	pH	OM	TP	TK	AP	AK
2023	TS	CK	8.18 ± 0.02Ab	14.36 ± 0.89Bb	0.242 ± 0.008Ab	13.98 ± 0.47Aa	8.75 ± 0.31Aa	255.44 ± 8.84Aa
		PC	8.40 ± 0.14Aab	15.09 ± 0.65Bab	0.258 ± 0.009Ab	14.07 ± 0.44Ba	8.02 ± 0.27Ab	245.11 ± 8.34Aa
		PM	8.43 ± 0.05Aa	15.89 ± 0.68Bab	0.284 ± 0.009Aa	14.81 ± 0.50Aa	7.93 ± 0.26Ab	237.24 ± 8.96Aa
		PB	8.37 ± 0.06Aab	16.57 ± 0.54Ba	0.291 ± 0.010Aa	14.91 ± 0.44Aa	6.82 ± 0.24Ac	248.75 ± 8.81Aa
	FHS	CK	8.03 ± 0.03Bb	17.39 ± 0.65Ab	0.240 ± 0.007Ab	14.21 ± 0.49Aa	5.43 ± 0.19Bc	209.87 ± 6.89Ba
		PC	8.19 ± 0.08Bab	19.79 ± 0.74Aa	0.166 ± 0.005Bc	14.84 ± 0.44Aa	4.85 ± 0.16Bd	200.43 ± 8.27Bab
		PM	8.30 ± 0.11Aa	18.05 ± 0.69Ab	0.288 ± 0.011Aa	13.80 ± 0.51Ba	7.31 ± 0.28Ba	201.47 ± 7.42Bab
		PB	8.12 ± 0.04Bab	17.12 ± 0.49Ab	0.257 ± 0.011Bb	14.86 ± 0.52Aa	6.28 ± 0.22Bb	188.61 ± 6.48Bb
2024	TS	CK	8.19 ± 0.03Aa	15.25 ± 0.86Ba	0.258 ± 0.009Ab	14.68 ± 0.52Ba	8.47 ± 0.30Aa	247.69 ± 8.72Aa
		PC	8.19 ± 0.09Aa	14.51 ± 0.51Ba	0.265 ± 0.009Ab	14.47 ± 0.51Ba	8.18 ± 0.29Aa	250.52 ± 8.82Aa
		PM	8.26 ± 0.08Aa	15.26 ± 0.54Ba	0.292 ± 0.010Aa	15.19 ± 0.53Aa	8.09 ± 0.28Aa	240.74 ± 8.48Aa
		PB	8.21 ± 0.08Aa	16.01 ± 0.56Aa	0.299 ± 0.011Aa	15.24 ± 0.54Aa	6.93 ± 0.24Ab	254.39 ± 8.96Aa
	FHS	CK	8.04 ± 0.05Aa	18.48 ± 0.65Aa	0.253 ± 0.009Ab	15.05 ± 0.53Aa	5.26 ± 0.19Bc	203.54 ± 7.17Ba
		PC	8.00 ± 0.07Ba	18.91 ± 0.67Aa	0.170 ± 0.006Bc	15.22 ± 0.54Aa	4.92 ± 0.17Bc	203.24 ± 7.20Ba
		PM	8.14 ± 0.13Aa	17.34 ± 0.61Aab	0.296 ± 0.010Aa	14.20 ± 0.42Ba	7.42 ± 0.26Ba	204.57 ± 6.19Ba
		PB	7.97 ± 0.06Ba	16.50 ± 0.58Ab	0.265 ± 0.009Bb	15.20 ± 0.54Aa	6.38 ± 0.22Ab	193.28 ± 6.81Ba

Different lowercase letters in the same column for the same year and variety show significant difference between samples atα = 0.05 (Tukey HSD). Different uppercase letters in the same column for the same year and variety show significant difference between samples at different stage atα = 0.05 (two-tailed). PB: boundary zone of ERC-12; PM: intermediate zone of ERC-12; PC: central zone of ERC-12. TS, the tillering stage. FHS, the full heading stage. OM, the soil organic matter. TP, the soil total phosphorus content. TK, the soil total potassium content. AP, the soil available phosphorus content. AK, the soil available potassium content. CK, the traditional rice-crab co-culture model. ERC-12, the optimized rice-crab co-culture model. Mean ± SD, n=3.

**Table 4:** The soil physicochemical properties of YF47.

Years	Stages	Treatments	pH	OM	TP	TK	AP	AK
2023	TS	CK	7.64 ± 0.16Aa	13.28 ± 0.55Ba	0.224 ± 0.008Ab	13.13 ± 0.40Aa	8.10 ± 0.30Aa	238.13 ± 8.57Aa
		PC	7.38 ± 0.17Aa	13.41 ± 0.65Ba	0.257 ± 0.008Aa	13.65 ± 0.50Aa	7.58 ± 0.30Aab	231.96 ± 9.60Aa
		PM	7.37 ± 0.23Aa	13.41 ± 1.07Aa	0.259 ± 0.022Aa	13.43 ± 0.97Aa	7.78 ± 0.64Aab	228.99 ± 16.87Aa
		PB	7.40 ± 0.24Aa	14.16 ± 0.63Aa	0.257 ± 0.013Aa	13.69 ± 0.65Aa	6.78 ± 0.27Ab	245.71 ± 11.56Aa
	FHS	CK	7.53 ± 0.16Aa	16.33 ± 1.34Aa	0.218 ± 0.019Aa	13.18 ± 1.16Aa	5.08 ± 0.44Bb	195.21 ± 15.96Ba
		PC	7.07 ± 0.19Aa	16.27 ± 0.70Aa	0.153 ± 0.007Bb	13.26 ± 0.77Aa	4.67 ± 0.21Bc	190.61 ± 10.02Ba
		PM	7.28 ± 0.11Aa	15.40 ± 1.17Aa	0.259 ± 0.022Aa	12.26 ± 0.99Aa	7.28 ± 0.61Aa	200.63 ± 15.78Aa
		PB	7.37 ± 0.09Aa	14.37 ± 0.90Aa	0.230 ± 0.016Ba	13.64 ± 0.90Aa	6.12 ± 0.36Bb	185.16 ± 11.30Ba
2024	TS	CK	7.61 ± 0.17Aa	14.11 ± 0.51Ba	0.235 ± 0.009Aa	13.86 ± 0.50Aa	7.87 ± 0.29Aa	230.40 ± 8.38Aa
		PC	7.56 ± 0.27Aa	14.20 ± 0.60Ba	0.272 ± 0.011Aa	14.44 ± 0.61Aa	7.33 ± 0.31Aab	223.74 ± 9.41Aa
		PM	7.50 ± 0.27Aa	14.31 ± 1.13Ba	0.272 ± 0.021Aa	14.25 ± 1.12Aa	7.53 ± 0.59Aab	221.89 ± 17.50Aa
		PB	7.69 ± 0.27Aa	15.07 ± 0.68Aa	0.270 ± 0.012Aa	14.40 ± 0.65Aa	6.55 ± 0.29Ab	238.34 ± 10.74Aa
	FHS	CK	7.53 ± 0.31Aa	17.39 ± 1.49Aa	0.232 ± 0.020Aa	13.79 ± 1.18Aa	4.92 ± 0.42Bbc	189.56 ± 16.27Ba
		PC	7.32 ± 0.26Aa	17.31 ± 0.85Aa	0.161 ± 0.008Bb	13.83 ± 0.68Aa	4.52 ± 0.22Bc	184.87 ± 9.14Ba
		PM	7.58 ± 0.35Aa	16.41 ± 1.28Aa	0.272 ± 0.021Aa	12.87 ± 1.00Aa	7.02 ± 0.55Aa	193.76 ± 15.11Aa
		PB	7.40 ± 0.34Aa	15.27 ± 0.98Aa	0.242 ± 0.015Aa	14.36 ± 0.92Aa	5.93 ± 0.38Ab	179.79 ± 11.52Ba

Different lowercase letters in the same column for the same year and variety show significant difference between samples atα = 0.05 (Tukey HSD). Different uppercase letters in the same column for the same year and variety show significant difference between samples at different stage atα = 0.05 (two-tailed). PB: boundary zone of ERC-12; PM: intermediate zone of ERC-12; PC: central zone of ERC-12. TS, the tillering stage. FHS, the full heading stage. OM, the soil organic matter. TP, the soil total phosphorus content. TK, the soil total potassium content. AP, the soil available phosphorus content. AK, the soil available potassium content. CK, the traditional rice-crab co-culture model. ERC-12, the optimized rice-crab co-culture model. Mean ± SD, n=3.

**Figure 5:**
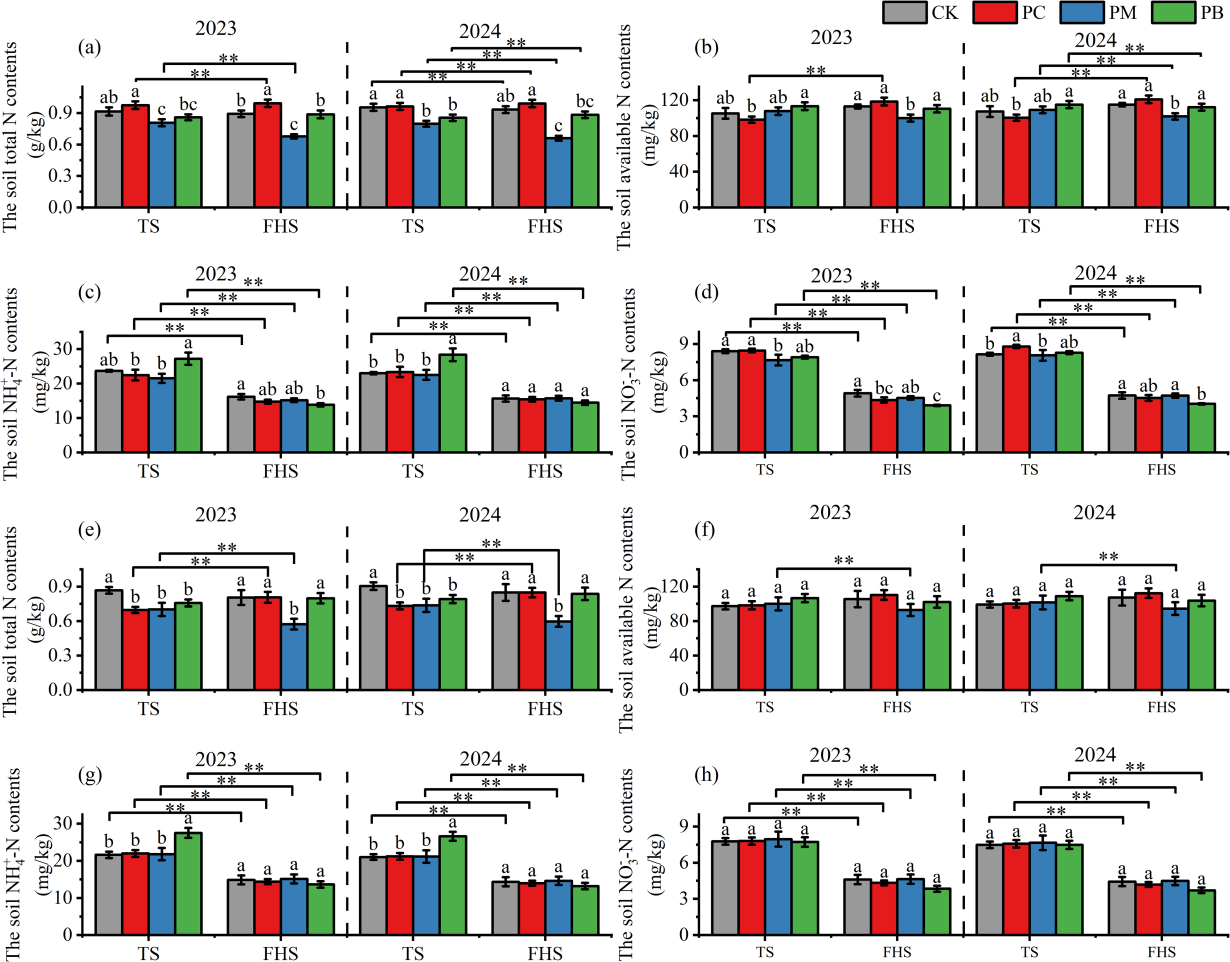
The contents of various forms of nitrogen in soil at different stage. **(a–d)** indicate YJ939. **(e–h)** indicate YF47. Different lowercase letters in the same stages show significant difference between samples at α = 0.05 (Tukey HSD). ** show significance difference between samples at α = 0.05 (two-tailed). PB: boundary zone of ERC-12; PM: intermediate zone of ERC-12; PC: central zone of ERC-12. TS, the tillering stage. FHS, the full heading stage. MS, the maturity stage. CK, the traditional rice-crab co-culture model. ERC-12, the optimized rice-crab co-culture model. Three biological replicates and the average deviation is used for the error line.

Analysis of TN, AN, $NO_{3}^{-}-N$ and $NH_{4}^{+}-N$ contents across treatments (**Figure 5**) showed that TN and AN contents varied slightly between the full tillering and full heading stage, whereas $NO_{3}^{-}-N$ and $NH_{4}^{+}-N$ contents differed significantly. For YJ939, TN content in the PM zone was significantly higher at the full tillering stage than at the full heading stage; AN content in the PC zone was significantly lower at the full tillering stage than at the full heading stage. For YF47, as rice growth progressed from full tillering to full heading, TN content in the PC zone increased significantly, while TN and AN contents in the PM zone decreased significantly. For both varieties, $NH_{4}^{+}-N$ content in the PB zone weas significantly higher than in the PM and PC zones at the full tillering stage, but significantly lower at the full heading stage. Additionally, $NO_{3}^{-}-N$ contents at the full tillering stage were significantly higher than those at the full heading stage for both YJ939 and YF47.

### 3.4 Soil nitrogen cycling functional gene

**Figure 6:**
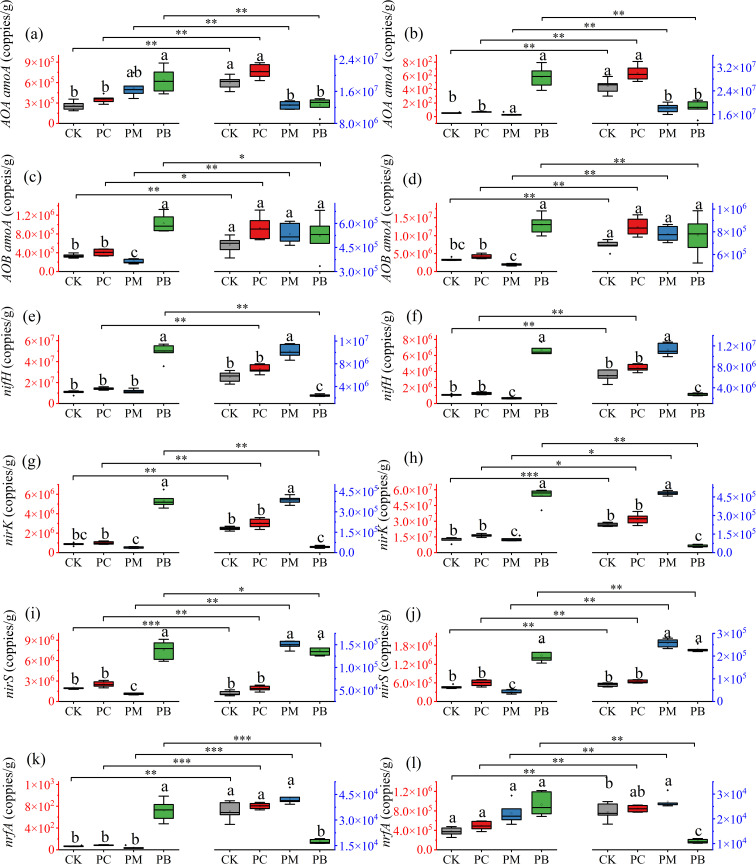
Abundance of the functional genes in soil of different stages of YJ939. **(a, c, e, g, i, k)** indicate the abundance of the functional genes in 2023. **(b, d, f, h, j, l)** indicate the abundance of the functional genes in 2024. The red axis indicates tillering stage of rice. The blue axis indicates full heading stage of rice. Different lowercase letters in the same stage show significant difference between samples at α = 0.05 (Tukey HSD). *, ** and *** show significance difference between samples at α = 0.05, 0.01 and 0.001 (two-tailed). PB: boundary zone of ERC-12; PM: intermediate zone of ERC-12; PC: central zone of ERC-12. CK, the traditional rice-crab co-culture model. ERC-12, the optimized rice-crab co-culture model. Six biological replicates and the average deviation is used for the error line.

**Figure 7:**
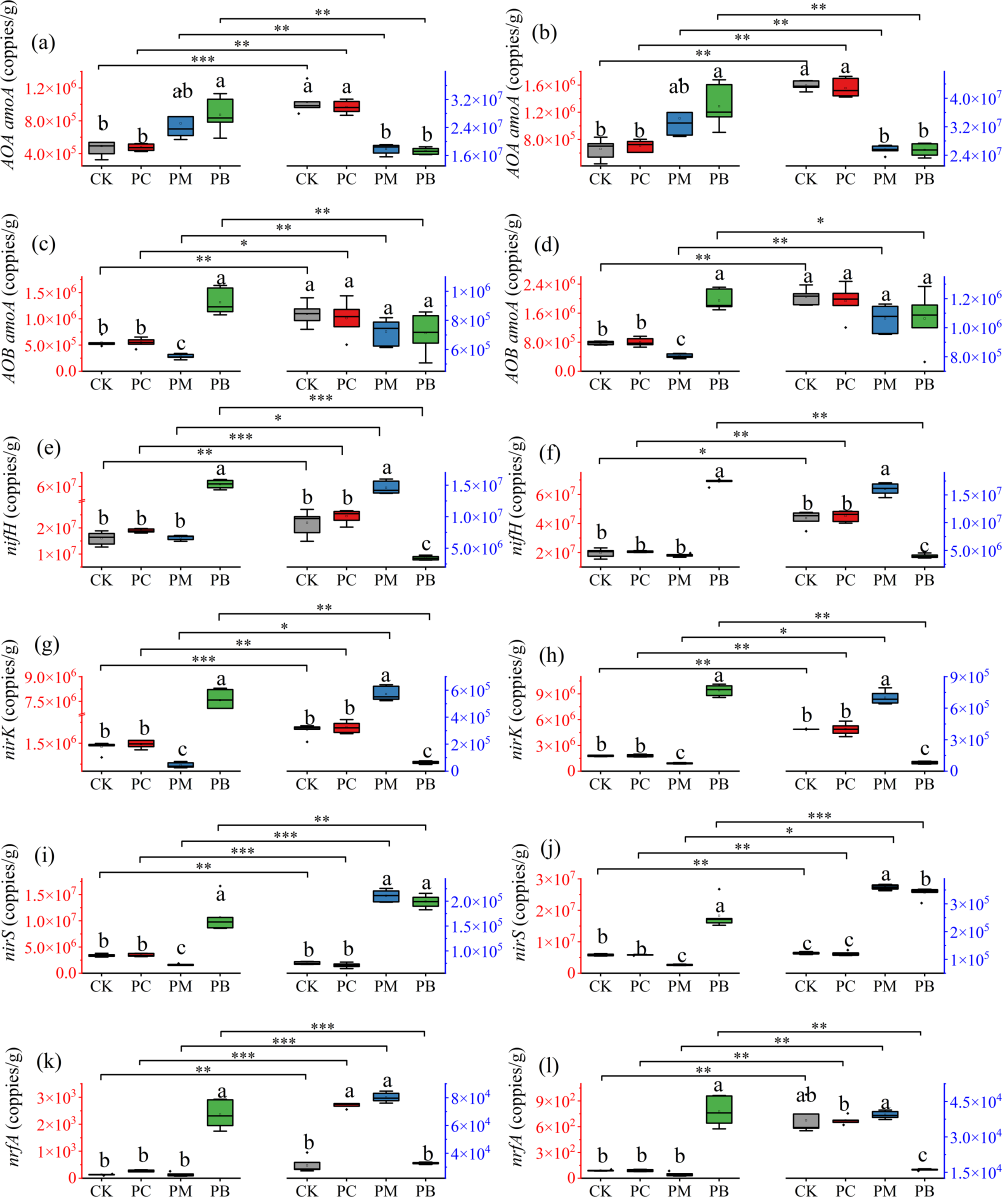
Abundance of the functional genes in soil of different stages of YF47. **(a, c, e, g, i, k)** indicate the abundance of the functional genes in 2023. **(b, d, f, h, j, l)** indicate the abundance of the functional genes in 2024. The red axis indicates tillering stage of rice. The blue axis indicates full heading stage of rice. Different lowercase letters in the same stage show significant difference between samples at α = 0.05 (Tukey HSD). *, ** and *** show significance difference between samples at α = 0.05, 0.01 and 0.001 (two-tailed). PB: boundary zone of ERC-12; PM: intermediate zone of ERC-12; PC: central zone of ERC-12. CK, the traditional rice-crab co-culture model. ERC-12, the optimized rice-crab co-culture model. Six biological replicates and the average deviation is used for the error line.

In this study, we used qPCR-based expression analysis to investigate differences in the expression levels of soil nitrogen cycling genes in the soil across treatments (**Figure 6** and **Figure 7**). The results revealed that the expression levels of soil nitrogen cycling functional genes significantly differed among stages, treatments, varieties and years. At the full tillering stage, the expression levels of the *AOA amoA*, *AOB amoA*, *nifH*, *nirK*, *nirS* and *nrfA* genes in the PB zone were significantly greater than those in all other treatments. Overall, the expression levels of nitrogen cycling functional genes at the full heading stage differed significantly from those at the full tillering stage. Specifically, the expression levels of *AOA amoA* in CK and PC were significantly higher than those of PM and PB, while the expression levels of *AOB amoA* showed no significant difference among treatments. The expression levels of the *nifH* and *nirK* in the PM zone were greater than those in CK and PC, whereas their expression levels in the PB zone were lower than those in CK and PC. The expression levels of *nirS* in PM and PB showed no significant difference between them but were significantly greater than those in CK and PC. In YF47, the expression levels of *nrfA* in CK, PC and PM show no significant difference among themselves but were significantly greater than those in PB. In YJ939, the expression levels of *nrfA* across treatments were similar to those in YF47, except that the levels in CK and PB showed no significant difference in 2023. These results indicate that soil microbial activity significantly differed among the zones of ERC-12.

### 3.5 Correlation analysis

To further investigate the drivers of yield differences across treatments, we performed correlation analyses for all parameter (**Table 5**). The results showed that effective panicle number per hill was significantly correlated with ADM and nitrogen accumulation at both the full tillering and maturity stage (p < 0.01). The spikelets per panicle were significantly correlated with ADM and nitrogen accumulation at the full heading stage (p < 0.01). The 1000-grain weight was significantly positively correlated with ADM and nitrogen accumulation at the full heading stage (p < 0.01) and with nitrogen accumulation at the maturity stage (p < 0.05). The seed setting rate and yield were significantly positively correlated with ADM and nitrogen accumulation across all growth stages (p < 0.01).

**Table 5:** The correlation analysis among yield, yield components, and aboveground dry matter and nitrogen contents of rice.

Yield and yield components Dry matter and nirtrogen content	Effective panicles	Spikelets numbers	1000-grains weight	Seed setting rate	Yield
TS-ADM	0.943^**^	-0.466^**^	-0.347^**^	0.591^**^	0.586^**^
FHS-ADM	0.100	0.512^**^	0.632^**^	0.608^**^	0.697^**^
MS-ADM	0.616^**^	0.113	0.247	0.858^**^	0.881^**^
TS-N	0.781^**^	-0.140	0.006	0.737^**^	0.815^**^
FHS-N	-0.058	0.611^**^	0.646^**^	0.430^**^	0.502^**^
MS-N	0.551^**^	0.180	0.321^*^	0.855^**^	0.888^**^

* and ** show significant correlations at α = 0.05 and 0.01, respectively (two-tailed). TS, the tillering stage. FHS, the full heading stage. MS, the maturity stage. ADM, the aboveground dry matter of rice. N, the nitrogen content of aboveground rice.

Correlation analyses were performed between ADM and nitrogen accumulation during different periods and various soil nitrogen forms (**Table 6**). In general, the AN and $NH_{4}^{+}-N$ contents at the full tillering stage were significantly positively correlated with ADM and nitrogen accumulation across all growth stages (p < 0.01), with significant positive correlations also observed for ADM increases from full tillering to full heading (p < 0.01) and nitrogen accumulation increases from full heading to maturity (p < 0.05). At the full heading stage, TN and AN contents were mostly positively correlated with ADM and nitrogen accumulation across all stages, whereas the $NH_{4}^{+}-N$ and $NO_{3}^{-}-N$ contents showed predominantly negative correlation with these parameters.

**Table 6:** The correlation analysis among soil nitrogen contents, aboveground dry matter and nitrogen contents of rice, and the increment of dry matter and nitrogen content of rice in different stages.

Dry matter and nirtrogen content Nigrogen component		TS		FHS		MS		TS-FHS		FHS-MS	
		ADM	N	ADM	N	ADM	N	ADM	N	ADM	N
TS	TN	0.227	0.333^*^	0.115	0.016	0.334^*^	0.266	0.640^**^	0.554^**^	-0.466^**^	-0.384^**^
	AN	0.304^*^	0.317^*^	0.302^*^	0.091	0.430^**^	0.409^**^	0.480^**^	0.356^*^	-0.177	0.044
	NH_4_^+^-N	0.339^**^	0.534^**^	0.636^**^	0.372^**^	0.668^*^	0.683^**^	0.553^**^	0.229	0.088	0.290^*^
	NO_3_^–^N	-0.033	0.066	-0.015	-0.157	0.109	0.073	0.528^**^	0.574^**^	-0.467^**^	-0.341^*^
FHS	TN	0.374^**^	0.563^**^	0.303^*^	0.238	0.523^**^	0.530^**^	0.661^*^	0.457^**^	-0.274	-0.159
	AN	0.265	0.430^**^	0.158	0.102	0.329^*^	0.359^**^	0.554^**^	0.448^**^	-0.325^*^	-0.220
	NH_4_^+^-N	-0.299^*^	-0.276	-0.413^**^	-0.291^*^	-0.373^**^	-0.328^*^	0.138	0.347^*^	-0.481^**^	-0.505^**^
	NO_3_^–^N	-0.400^**^	-0.452^**^	-0.557^**^	-0.384^**^	-0.562^**^	-0.532^**^	-0.137	0.174	-0.393^**^	-0.532^**^

* and ** show significant correlations at α = 0.05 and 0.01, respectively (two-tailed). TS, the tillering stage. FHS, the full heading stage. MS, the maturity stage. ADM, the aboveground dry matter of rice. N, the nitrogen content of aboveground rice. TN, the soil total nitrogen content. AN, the soil available nitrogen content. $NH_{4}^{+}-N$ , the soil ammonium nitrogen content. $NO_{3}^{-}-N$ , the soil nitrate nitrogen content.

**Figure 8:**
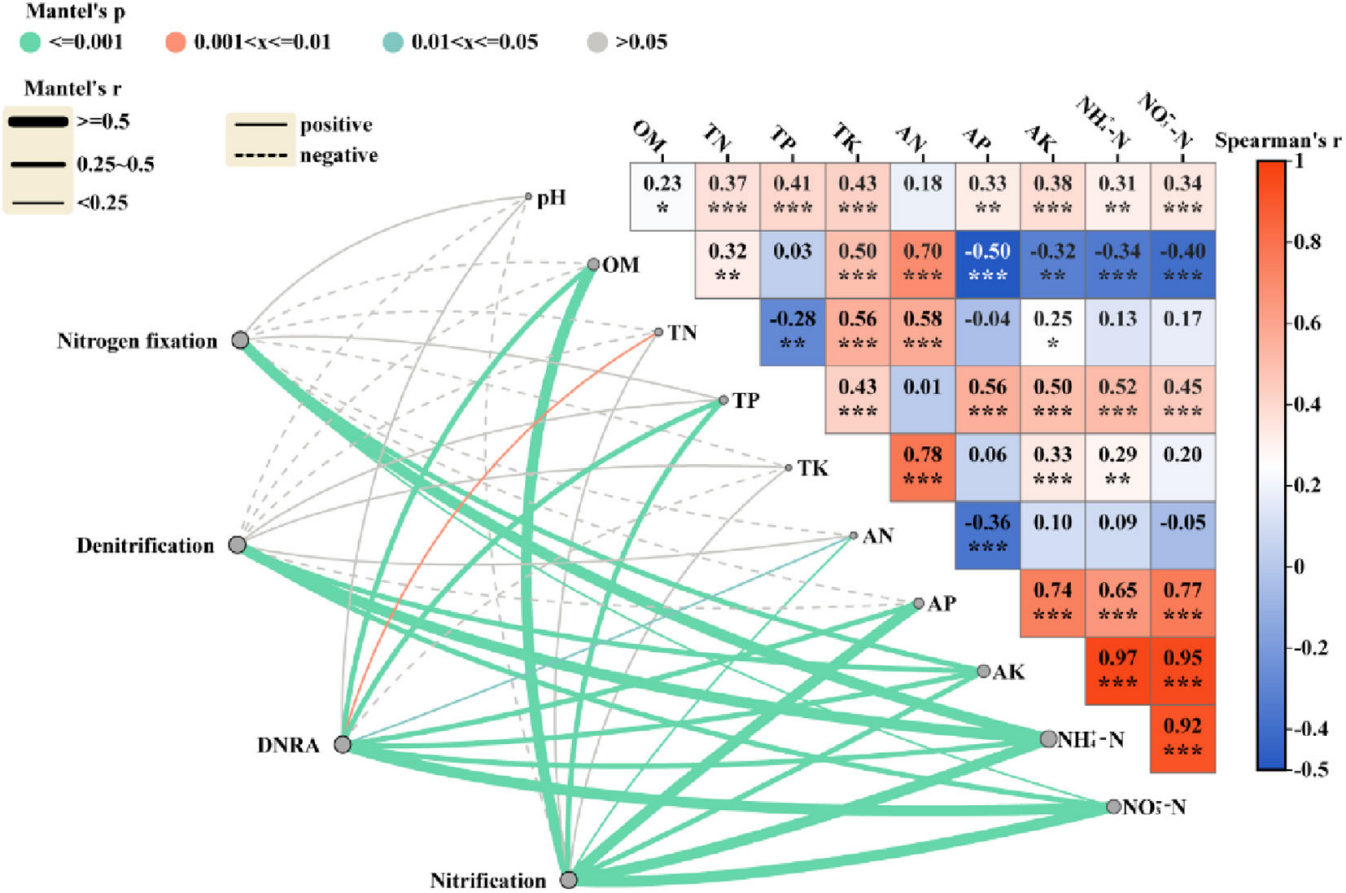
The soil physicochemical properties driving the soil nitrogen cycle gene abundance. *, ** and *** show significance at *p* < 0.05, 0.01 and 0.001, respectively (two-tailed). Nitrogen fixation includes *nifH*; Denitrification includes *nirK* and *nirS*; Nitrification includes *AOA amoA* and *AOB amoA*; DNRA includes *nrfA*. OM, the soil organic matter. TN, the soil total nitrogen content. TP, the soil total phosphorus content. TK, the soil total potassium content. AN, the soil available nitrogen content. AP, the soil available phosphorus content. AK, the soil available potassium content. NH_4_^+^-N, the soil ammonium nitrogen content. NO_3_^–^N, the soil nitrate nitrogen content.

**Figure 9:**
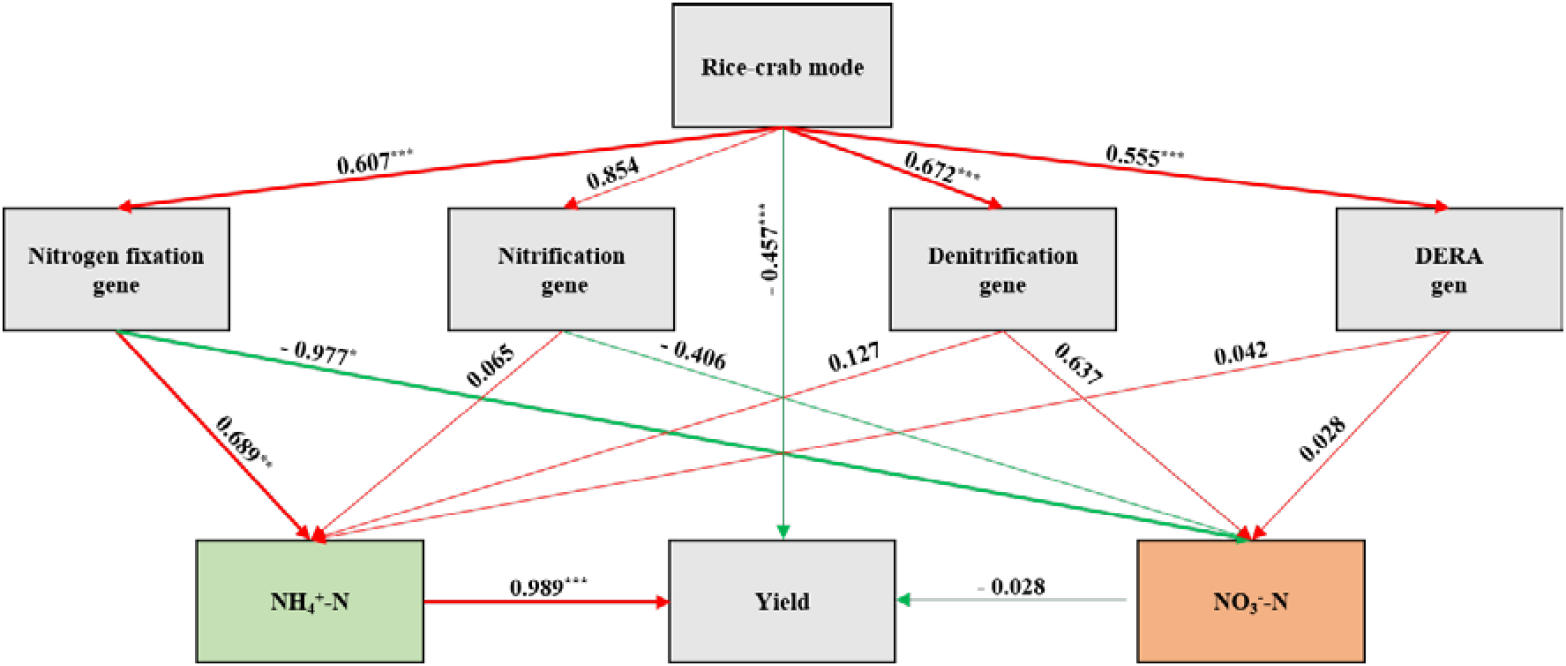
Structural Equation Modeling Path Analysis. The red line indicates a positive correlation; the green line indicates a negative correlation. *, **, *** show significance at *p* < 0.05, 0.01, 0.001, respectively. Multi-group analysis show significant between-group differences where Δχ² test *p* < 0.05. Nitrogen fixation includes *nifH*; Denitrification includes *nirK* and *nirS*; Nitrification includes *AOA amoA* and *AOB amoA*; DNRA includes *nrfA*. $NH_{4}^{+}-N$ , the soil ammonium nitrogen content. $NO_{3}^{-}-N$ , the soil nitrate nitrogen content.

We established relationships between the expression levels of soil nitrogen cycling functional genes and contents of various nitrogen forms (**Figure 8**), revealing that the $NH_{4}^{+}-N$ and $NO_{3}^{-}-N$ contents were significantly positively correlated with the pH, TP and AP contents, but negatively correlated with OM content. The nitrogen**–**fixation, nitrification, denitrification and dissimilatory nitrate reduction to ammonium (DNRA) functional genes were significantly associated with AK, $NH_{4}^{+}-N$ and $NO_{3}^{-}-N$ contents. The PLS-SEM analysis revealed the pathway by which the rice**–**crab coculture model affects yield through regulating soil nitrogen cycle (**Figure 9**). The pathway was supported by two lines of evidence. One is, that crab activity enhances soil aeration, thereby promoting the expression of *nifH* gene (Li et al., 2024b). Another is, that $NH_{4}^{+}-N$ , as the main nitrogen source for rice, directly affects dry matter accumulation (**Table 5**). Using SmartPLS, 3000 bootstrapping tests showed that all pathways were statistically significant. The rice**–**crab coculture model promoted *nifH* expression (β=0.607, p<0.001), soil *nifH* expression positively affected $NH_{4}^{+}-N$ (β=0.689, p<0.01), and soil $NH_{4}^{+}-N$ content positively influenced yield (β=0.989, p<0.001). These results verify that *nifH-*mediated biological nitrogen fixation indirectly promotes yield. This consistent positive causal relationship indicates that regulating soil nitrogen-fixing functional genes and $NH_{4}^{+}-N$ content.

## 4 Discussion

The Rice**–**crab coculture model is an ecologically sound cultivation model that integrates rice planting and crab breeding and can achieve efficient recycling and utilization of matter and energy by improving the biodiversity of paddies (Bao et al., 2022; Khoshnevisan et al., 2021). Due to this advantage, the rice**–**crab coculture model has substantia development potential and economically attractive in the single**–**cropping rice planting region in Northeast China.

### 4.1 Effects of the rice–crab coculture model on rice yield

The original purpose of the rice**–**crab coculture model was to increase economic benefits of paddy rice cultivation through crabs integration (Zhou et al., 1995). Although the production of crabs can increase economic returns, stable rice output remains critical for food security amid China’s population growth pressure (Chen et al., 2024). In this study, we compared the rice yields between CK and ERC-12, and investigated differences in ERC-12 yield components relative to CK based on the spatial distribution of rice plants. The marginal effect in ERC-12 increased the yield per plant in the PB zone by 9% (**Figure 2**), consistent with Ma et al. (2023), who reported that marginal row dominance enhances rice photosynthetic efficiency. However, the PM zone exhibited a yield decline due to nutrient competition, aligning with Bashir et al. (2021), who observed nitrogen deficiency in high-density planting areas of the rice**–**crab coculture model. Biological nitrogen fixation partially compensated for yield losses (approximately 2%), differing from the yield maintenance mechanism under the traditional fertilization regimes (Hu et al., 2020). Yield components are important determinants of rice yield (Huang et al., 2011; Kesh and Khan, 2023). In the ERC-12 model, the PB zone, due to its proximity to empty rows, benefits from enhanced light exposure, nutrient availability, and growing space, thereby inducing a marginal effect (Dong et al., 2025). Our statistical analysis reveals that this marginal effect translates into significant increases in key yield components within the PB zone. Specifically, the effective panicle number per hill and seed setting rate in the PB zone are significantly higher than in the PM and PC zones (p<0.05). Compared to the CK, these values increase by 5.57%-12.17% and 3.60%-7.70%, respectively. Notably, the PB zone’s contribution to the overall ERC-12 yield is substantial, ranging from 40.6% to 42.8%, which offsets the yield reduction in the PM zone (24.5%-26.3%). This is similar to results of Jiang et al. (2024) and Liu et al. (2024), who confirmed that high effective panicle number and high seed setting rate are critical for achieving high yield. Thus, although the marginal row effect of PB compensates for the reduced planting density in ERC-12, empty rows remain the primary factor constraining yield. It should be noted that our previous research provided ERC-12 delivered a greater overall economic return than CK (Ma et al., 2023). Its unique empty rows enhanced crabs survival, leading to higher crab yields. This increase in crabs production compensates for the loss in rice yield and boosts economic returns by approximately CNY 3,000 per hectare (S3).

The aboveground dry matter of rice not only positively correlates with nitrogen accumulation but also determines the amount of non-structural carbohydrates available to grains, which affects rice yield (Jiang et al., 2024). We found that the ADM and nitrogen accumulation in rice aboveground tissues were significantly correlated with rice yield. In ERC-12, the PB had the highest ADW, while the PM zone had the lowest, which corresponded to the results of the yield components. Fageria et al. (2008) reported the yield characteristics of 12 rice genotypes, noting an extremely significant positive quadratic correlation between stem dry matter accumulation and grain yield. Zhao et al. (2024) reported that a transplanting density of 333000 hills/ha, increasing the nitrogen fertilizer application increased dry matter weight and stem/sheath transport capacity, thereby increasing rice yield. Apart from being affected by the marginal effect of the PB, rice in PM zone remained disadvantaged in terms of nutrient supply during each period, which may explain its low dry matter accumulation. Therefore, ERC-12 yield can be further increased by: (1) precisely positioning and quantitatively controlling the base fertilizer release in the PM zone; (2) optimizing rice population structure in the PB, PM and PC zones; (3) enhancing dry matter accumulation during different periods.

### 4.2 Effects of the rice–crab coculture model on soil properties

At present, paddy coculture systems mainly include rice**–**shrimp, rice**–**fish, rice**–**crab, rice**–**turtle and rice**–**duck coculture models (Bao et al., 2022). Compared with rice monoculture, the coculture models alter soil physicochemical properties due to the activities of aquatic animals (Miao et al., 2022). Considering the living habits of crabs in rice**–**crab coculture model (Li et al., 2022; Zhao and Yang, 2024), we analyzed soil physicochemical properties in CK and the PC, PM and PB zones of ERC-12 at the full tillering and full heading stages. The results revealed that the soil physicochemical properties dynamically changed with rice growth, with significant differences among the PC, PM and PB zones of ERC-12. The PM and PB zones of ERC-12 had relatively high contents of OM, TP and TK in the early rice growth stage, whereas the opposite was observed in the middle and late stages. This is similar to the results of Li et al. (2024b), who reported that crabs agitate the water, the water**–**soil interface, and soil during their activities, increasing soil**–**air contact. This affected microbial richness and diversity in the topsoil, indirectly altering soil physicochemical properties. Ren et al. (2023) also reported noted that rice**–**fish coculture enhances soil retention of organic carbon, nitrogen and phosphorus. Given their nocturnal behavior, crabs select suitable hiding and sheltering sites (Li et al., 2022a). We hypothesized that, owing to the low ambient temperatures that prevailed during the period from the transplanting stage to the full tillering stage of rice, the crabs mainly gathered in deep water area of the ditch of the blank row of ERC-12 and immediately around this area; in the full heading stage, owing to the high air temperatures, the crabs mainly gathered in the PC zone of ERC-12 because the canopy blocked the high-temperature radiation of the sun. In the active areas, crab feces and mud agitated by the crabs resulted in changes in the soil physicochemical properties (Jiang et al., 2021).

Rice is an ammonium-utilizing plant. Therefore, increasing the ammonium nitrogen content in paddies promotes nitrogen accumulation in rice (Li et al., 2023). In the rice**–**crab coculture model, crab feed serves as both a direct and indirect additional nitrogen source, of which 59.1% enters the soil microbial metabolic cycle and 7.6% is absorbed and utilized by rice (Hu et al., 2020; Li et al., 2021). We found that TN, AN, $NH_{4}^{+}-N$ and $NO_{3}^{-}-N$ contents dynamically changed across rice growth stages, consistent with the activity patterns of crabs (Li et al., 2022). Bashir et al. (2021) reported that, compared with rice monoculture, the rice**–**crab coculture model significantly increased the soil microbial nitrogen content by 18.1% and ammonium nitrogen content by 728.9%. Li et al. (2008) confirmed that the rice**–**fish and rice**–**duck coculture models reduced soil NH_3_ volatilization by 3.21 kg N/ha and 1.41 kg N/ha, respectively, relative to monoculture, thereby improving nitrogen use efficiency. The sites where crabs were active provided more $NH_{4}^{+}-N$ to rice, facilitating plant dry matter accumulation. Therefore, in terms of soil nutrient supply, the different environments formed in the PC, PM and PB zones of ERC-12 improved the soil nitrogen use efficiency, which provided a larger nitrogen “source” for rice in the vegetative growth stage, and thus improved the potential for high yields of rice.

### 4.3 Effects of the rice–crab coculture model on soil nitrogen cycling functional genes

Soil nitrogen cycling functional genes play important roles in the key processes of microbial nitrogen fixation, nitrification, denitrification and nitrate dissimilatory reduction (Li et al., 2019; Sun et al., 2015), reflecting the nitrogen transformation process in soil to a certain extent. Our results revealed that the expression levels of nitrogen cycling functional genes differed among the different treatments and among the different stages. We found that in the PB zone of ERC-12, the expression levels of six soil nitrogen cycle functional genes were higher than those in other treatments during the tillering stage of rice, while the opposite was true during the full heading stage. Nitrogen fixation mediated by *nifH* and the dissimilatory nitrate reduction to ammonium (DNRA) controlled by *nrfA* are the dominant processes governing the soil nitrogen cycle in ERC-12. These two pathways counterbalance NH_4_^+^-N losses arising from nitrification (*AOA amoA* and *AOB amoA*) and denitrification (*nirK* and *nirS*), and concurrently increase soil $NH_{4}^{+}-N$ concentrations. This was consistent with the report by Li et al. (2024b) that crab activity enhances soil aeration and promotes the colonization of nitrogen-fixing bacteria. Luo et al. (2016) investigated soil nitrification with long-term straw return and reported that changes in the soil available phosphorus and available potassium contents significantly affected the *AOB amoA* expression level. Similarly, Tang et al. (2017) reported that the loss of phosphorus and potassium in soil reduced the expression levels of the *nifH* gene and reduced the nitrogen fixation ability of soil. Ding et al. (2019) revealed that the expression levels of soil nitrogen cycling functional genes are usually affected by the activity and abundance of related soil microorganisms and are closely related to soil physicochemical properties, fertilization management, vegetation types and environmental conditions. Furthermore, Li et al. (2022b) confirmed that factors affecting soil microbial activity, such as water management, nutrient content, and pH, regulate the expression levels of the *nirK* and *nirS* genes and affect the process of soil denitrification. In ERC-12, crabs had different activity areas at different growth stages of rice, which caused changes in the richness and diversity of soil microbial communities and the physicochemical properties of the soil (Ma et al., 2024b). There, we postulate that the metabolic and behavioral activities of crabs elevate soil AP and AK contents in actively zones, indirectly up-regulating *nifH* and *nrfA* expression and consequently increasing soil $NH_{4}^{+}-N$ . Moreover, bioturbation during crab locomotion disperses surface soil, enhancing the probability of contact between nitrogen gas and soil enriched in *nifH*-expression and thereby promoting nitrogen fixation. Path analysis revealed that *nifH* expression indirectly promoted yield (path coefficient 0.989, p<0.001) by increasing the $NH_{4}^{+}-N$ content (path coefficient 0.689, p<0.01), providing evidence for soil nitrogen fixation effect-nitrogen supply-yield formation.

Although the ERC-12 model mitigated some yield losses through soil nitrogen fixation, its environmental impacts remain a concern. In terms of greenhouse gas emissions, the burrowing and soil-stirring behaviors of crabs can disrupt soil aeration (Li et al., 2024b), promoting the activity of methanogens under waterlogged conditions and thereby increasing CH_4_ emissions (Bashir et al., 2021, 2025). In terms of soil health, the synergistic effects of post-harvest rice straw incorporation and crab residue decomposition have not been evaluated, and whether the elevated expression of the nitrogen fixation gene *nifH* would suppress the diversity of other microbial communities requires further investigation. In terms of feed input, crabs have a nitrogen utilization rate of approximately 35% for feed nitrogen (Hu et al., 2020), meaning that 65% of the nitrogen enters the soil or water in the form of uneaten feed and feces, posing a risk of eutrophication in surrounding water bodies. Additionally, the present study did not quantify crab density or activity across ERC-12 zones. Crab behavior is known to influence soil aeration and nutrient redistribution, which may have indirectly affected microbial nitrogen fixation and ammonium supply in PB and PM zones. Future research should incorporate direct measurements of crab activity to clarify its role in mediating spatial heterogeneity in soil nitrogen dynamics and rice yield.

This study focused on the microbial-driven nitrogen cycling in the rice-crab system but did not quantify nitrogen use efficiency (NUE) or the contribution rate of biological nitrogen fixation. Future improvements could be made through the following approaches: Firstly, using ^15^N isotope labeling to determine the actual contribution of biological nitrogen fixation to rice nitrogen; Secondly, calculating system NUE = (rice nitrogen uptake/total nitrogen input + biological nitrogen fixation) ×100%; Thirdly, combining field nitrogen balance models to assess the nitrogen use advantages of ERC-12; Lastly, measuring CH_4_ and N_2_O fluxes using static chamber methods. These quantitative indicators will provide more precise scientific basis for the sustainability of rice-crab co-culture model. Additionally, factors such as rice pests and diseases, extreme weather, light availability, water movement, and soil temperature may also affect the results of this study. Nevertheless, this study still offers a new perspective for exploring the rice yield potential of different rice-crab co-culture models.

## 5 Conclusion

The optimized rice**–**crab coculture model (ERC-12) enhanced soil nitrogen fixation in the boundary rows of the rice planting are through the empty row, improved ammonium nitrogen supply efficiency, promoted rice aboveground dry matter accumulation during different growth periods, and increased the boundary yield advantage, offsetting the loss from the empty row area by 2%. However, nutrient competition and insufficient microbial activity in the PM zone of ERC-12 remained key factors limiting yield improvement. In the future, the rice population structure in the PB, PM and PC zones of ERC-12 can be optimized by directional and fixed-point slow-release fertilizer application—especially by elevating nutrient supply in the PM zone—which would further increase the yield potential of ERC-12 and result in high yields of both rice and crab.

## Data Availability

The raw data supporting the conclusions of this article will be made available by the authors, without undue reservation.
