# Supplementary material for: Spatial yield gains in empty-row optimized rice–crab co-culture are linked to nifH-driven nitrogen compensation in border rows
Source: Front Plant Sci. 2025 Aug 15;16:1607596. doi: 10.3389/fpls.2025.1607596 (PMC12394533; doi:10.3389/fpls.2025.1607596)
Supplement: Supplementary file 1 [file Table1.docx]

**S1.**

| **Table S1. Rice yield per plant in ERC-12 coculture model.** | |
| --- | --- |
| Site | Weight (g) |
| R1 | 58.23±3.35a |
| R2 | 57.19±1.08a |
| R3 | 40.25±1.54c |
| R4 | 38.35±3.11c |
| R5 | 49.35±1.25b |
| R6 | 50.31±4.39b |
| Note: In ERC-12, the single-hill rice from the marginal to the central is signed R1, R2, R3, R4, R5 and R6. Different letters in the same column for the same year indicate a significant difference at a level of 0.05 (one-way ANOVA followed by Tukey’s HSD post-hoc test). | |

**S2.**

| **Table S2. qPCR parameters** | | |
| --- | --- | --- |
| Type of PCR | Steps | Temperature and Time Parameters |
| Target gene PCR amplification (plasmid cloning stage) | Pre-denaturation | 94℃, 5 min |
|  | Cycling (30 cycles) | 94℃ denaturation for 30 sec  55℃ annealing for 30 sec  72℃ extension for 30 sec |
|  | Final extension | 72℃, 10min |
| Colony PCR for identifying positive clones | Pre-denaturation | 94℃, 5 min |
|  | Cycling (30 cycles) | 94℃ denaturation for 30 sec  55℃ annealing for 30 sec  72℃ extension for 30 sec |
|  | Final extension | 72℃, 10min |
| Real time PCR detection | Pre-denaturation | 95℃, 10 min |
|  | Cycling (40 cycles) | 95℃ denaturation for 15 sec  60℃ annealing for 1 min |
|  | Melting curve stage | 95℃ for 15 sec  60℃ for 1 min  95℃ for 30 sec  60℃ for 15 sec  Slowly heating from 60℃ to 99℃ (Ramp rate is 0.05℃/sec) |

| **Table S3. Primers sequence** | | |
| --- | --- | --- |
| Primer full name | Forward primer sequence (5` to 3`) | Reverse primer sequence (5` to 3`) |
| *AOA amoA* | GACTACATMTTCTAYACWGAYTGGGC | GGKGTCATRTATGGWGGYAAYGTTGG |
| *AOB amoB* | GGGGTTTCTACTGGTGGT | CCCCTCKGSAAAGCCTTCTTC |
| *nifH* | AAAGGYGGWATCGGYAARTCCACCAC | TTGTTSGCSGCRTACATSGCCATCAT |
| *nirK* | ATYGGCGGVCAYGGCGA | GCGTCGATCAGRTTRTGGTT |
| *nirS* | GTSAACGTSAAGGARACSGG | GASTTCGGRTGSGTCTTGA |
| *nrfA* | CARTGYCAYGTBGARTA | TWNGGCATRTGRCARTC |

**S3.**

| **Table S4. Average yields of agricultural products per hectare in CK and ERC-12.** | | | | | | |
| --- | --- | --- | --- | --- | --- | --- |
| Varieties | Treatments | Rice yield (kg) | Male crabs | | Female crabs | |
|  |  |  | Number | Weight (kg) | Number | Weight (kg) |
| YJ939 | CK | 10607.7 | 1750 | 168.00 | 1575 | 119.70 |
|  | ERC-12 | 10145.6 | 1893 | 232.84 | 1616 | 135.74 |
| YF47 | CK | 10762.3 | 1695 | 161.53 | 1603 | 123.82 |
|  | ERC-12 | 10260.4 | 1887 | 230.35 | 1945 | 162.80 |
| Note: CK, the conventional rice**–**crab coculture model; ERC-12, the optimized 12**–**rows**–**cultivated**–**1**–**row**–**empty model. | | | | | | |

| **Table S5. Economic benefit analysis of CK and ERC-12 (CNY/ha).** | | | | | | |
| --- | --- | --- | --- | --- | --- | --- |
| Treatments | Cost of rice production | Cost of crab production | Rive revenue | Crab revenue | Total revenue | Output-input ratio |
| CK | 10800 | 7800 | 35079 | 13810 | 30289 | 2.63 |
| ERC-12 | 10440 | 7800 | 33957 | 17692 | 33409 | 2.83 |
| Note: CK, the conventional rice**–**crab coculture model; ERC-12, the optimized 12**–**rows**–**cultivated**–**1**–**row**–**empty model. | | | | | | |
